# High salt diet stimulates gut Th17 response and exacerbates TNBS-induced colitis in mice

**DOI:** 10.18632/oncotarget.13783

**Published:** 2016-12-01

**Authors:** Yingfeng Wei, Chong Lu, Jianing Chen, Guangying Cui, Lin Wang, Tianming Yu, Yue Yang, Wei Wu, Yulong Ding, Lanjuan Li, Toshimitsu Uede, Zhi Chen, Hongyan Diao

**Affiliations:** ^1^ State Key Laboratory for Diagnosis and Treatment of Infectious Diseases, Collaborative Innovation Center for Diagnosis and Treatment of Infectious Diseases, The First Affiliated Hospital, College of Medicine, Zhejiang University, Hangzhou, Zhejiang, China; ^2^ Department of Orthopaedics, Sir Run Run Shaw Hospital, College of Medicine, Zhejiang University, Hangzhou, Zhejiang, China; ^3^ Department of Biochemistry, University of Washington, Seattle, Washington, USA; ^4^ Molecular Immunology, Institute for Genetic Medicine, Hokkaido University, Sapporo, Japan

**Keywords:** high salt diet, Th17 cells, regulatory T cells, inflammatory bowel diseases, intestinal immunity, Immunology and Microbiology Section, Immune response, Immunity

## Abstract

This study focuses on characterizing the effect of a high salt diet (HSD) on intestinal immunity and the risk of inflammatory bowel diseases (IBD). We found that mice on a HSD had an increased frequency of IL-17A producing cells in the intestinal lamina propria (LP) compared to mice on a normal diet (ND). Furthermore, most intestinal IL-17A producing cells were CD4^+^TCRβ^+^ cells. A HSD increased the LP T helper 17 (Th17) responses in both the small and large intestines but did not increase the Th17 response of other gut-associated lymphoid organ. Although, HSD did not change the percentage of regulatory T (Treg) cells, HSD significantly inhibit secretion of IL-10 and the suppressive function of Treg cells. Moreover, we found that HSD exacerbates trinitrobenzenesulfonic acid (TNBS) induced colitis, and Th17 response was significantly increased in the colonic LP of HSD TNBS-treated mice compared with the ND TNBS-treated mice. This study demonstrates that HSD stimulates the intestinal Th17 response but inhibits the function of Treg cells. Moreover, HSD exacerbates TNBS induced mice colitis, suggesting that HSD disrupts the intestinal immunity and increases the risk of IBD.

## INTRODUCTION

Poor eating habits contribute to various human illnesses and may be considered critical risk factors for diseases such as obesity and cardiovascular disease [1]. Dietary sodium chloride (NaCl) is relatively high in Western diets, as is consumption of processed and ‘fast foods’ [2, 3], which often contain 100 times the salt content of homemade foods [3, 4]. It has been well established that excess NaCl intake is closely linked to the development of cardiovascular disease and stroke [5, 6]. However, there is growing evidence that excess NaCl also affects the immune system, leading to an increased incidence of autoimmune diseases [7–9].

A recent study revealed that excess consumption of NaCl increases the number of monocyte in the human peripheral blood, and is accompanied by enhanced production of pro-inflammatory cytokines and reduced anti-inflammatory factors [10]. A high salt diet (HSD) in rats leads to interstitial Na^+^ accumulation in the skin, resulting in the activation of macrophages and increased density and hyperplasia of the lympho-capillary network [9]. Importantly, studies confirmed that even a modest increase in the concentration of NaCl markedly enhances interleukin (IL)-17-producing CD4^+^ helper T cells (Th17 cells) responses *in vitro*, as does a HSD *in vivo* [7, 8]. It has also been shown that, when compared to a normal salt diet (ND), a HSD increases the severity of experimental autoimmune encephalomyelitis (EAE) in mice accompanied by increased Th17 response [7, 8].

Th17 cells are a subset of CD4^+^ T helper cells that produce IL-17A, IL-17F, and IL-21 [11]. Among these cytokines, IL-17A is the most thoroughly studied and is considered the signature effector cytokine of Th17 cells [12]. TGF-β, IL-6, and IL-21 are cytokines that promote the differentiation of naive T cells into mature Th17 cells, while IL-23 is a growth and stabilization factor of Th17 cells [11]. Th17 cells participate in the host defense against bacterial and fungal pathogens, and are strongly associated with autoimmune diseases such as multiple sclerosis, rheumatoid arthritis, and inflammatory bowel diseases (IBD) [11, 13]. IBD is characterized by chronic relapsing inflammation that occurs in two major forms: Crohn's disease and ulcerative colitis. Th17 cells are present throughout the intestinal lamina propria (LP) [14] and studies have suggested that Th17 cells and Th17-associated molecules play a critical role in pathogenesis of IBD [15–17]. By example, up-regulation of IL-17A and IL-17F has a known pathogenic role in the development of IBD [15, 17–20]. Notably in animal models IL-17R knockout mice protected against the development of IBD [16]. The orphan nuclear receptor (RORγt) is the critical transcription factor of Th17 cells [11, 14], and RORγt has been reported to control the production of IL-17A and IL-17F, thereby modulating the pathogenicity of IBD mouse models [15, 20].

Previous studies have revealed that the incidence of IBD is closely related to dietary habits [21], with high fat intake increasing the risk of IBD, while a high fiber or fruit diet decreases the risk [21]. As the association between a HSD and IBD remains incompletely characterized, we investigate the effects of HSD on intestinal immunity and report the degree of colitis seen in a trinitrobenzenesulfonic acid (TNBS)-induced mouse model of IBD.

## RESULTS

### HSD selectively promote the intestinal LP IL-17A producing cells

**Figure 1:**
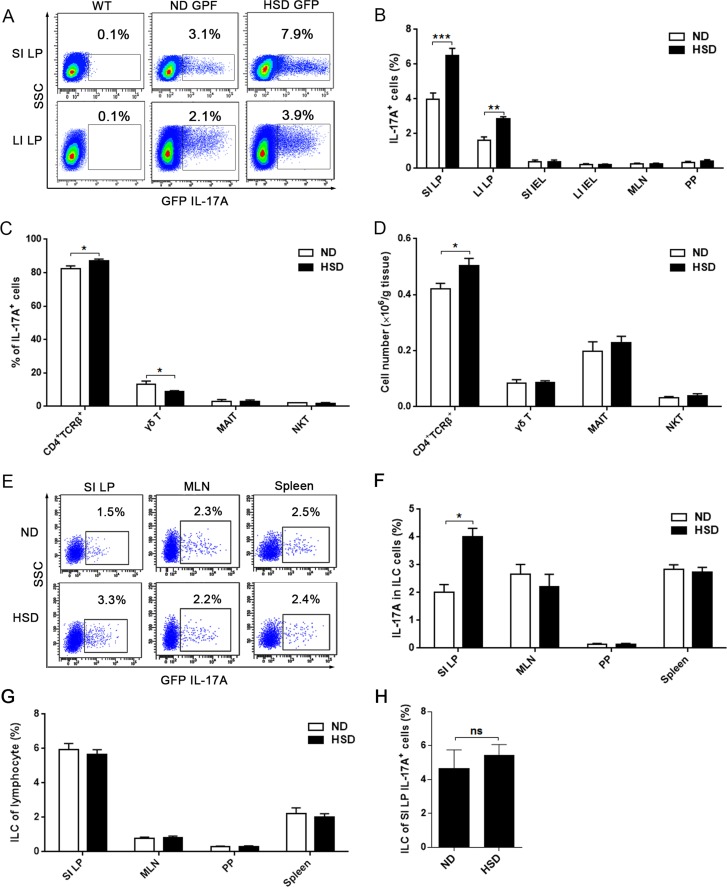
HSD stimulates IL-17A producing cells in intestinal lamina propria (LP) We isolated lymphocytes from small intestinal LP (SI LP), large intestinal LP (LI LP), small intestinal intraepithelial (SI IEL), large intestinal intraepithelial (LI IEL), mesenteric lymph node (MLN), and Peyer's patch (PP), the mice include ND WT mice and IL-17A-GFP mice that accept 3 weeks ND or HSD. Immediately following isolation, cells were incubated for 4 hr with PMA/Ionomycin and GolgiPlug, and analyzed by flow cytometry. **A.** Representative flow cytometric scatterplot of GFP IL-17A^+^ cells in the SI LP and LI LP, data were gated on lymphocytes. **B.** The proportion of GFP IL-17A^+^ cells within the lymphocytes obtained from different locations (*n* = 8). **C.** After gated on the SI LP IL-17A^+^ cell, the cell fraction of CD4^+^TCRβ^+^ cells, γδ T cells, MAIT cells and NKT cells was measured (*n* = 8). **D.** The absolute number of CD4^+^TCRβ^+^ cells, γδ T, MAIT and NKT cells of the SI LP, data were acquired by flow cytometry and is expressed as cell number per gram of the small intestine (n = 8). **E.** The representative flow cytometric scatterplot of GFP IL-17A production of ILCs (lineage^−^CD45^+^CD90.2^+^) from the SI LP, MLN and spleen cells, the gated program was showed in Supplementary Figure 2. **F.** Summative histogram of GFP IL-17A expression in ILCs (*n* = 6). **G.** The percentages of ILCs in the lymphocytes from SI LP, MLN, PP and spleen (*n* = 6). **H.** Of IL-17A producing cells, the percentage of ILCs in the SI LP was analyzed (*n* = 6). Data are expressed as mean ± SEM from three independent experiments.

To investigate the effects of a high salt diet on the intestines, IL-17A producing cells were analyzed from the lamina propria (LP) of the small (SI) and large (LI) intestines. In HSD mice, IL-17A producing cells were significantly increased in both small and large intestinal LP compared to those mice on ND (Figure 1A). In contrast to the LP, the proportion of IL-17A producing cells in other locations was very low (including the SI intraepithelial lymphocytes (IEL), LI IEL, MLN, and PP) (Figure 1B). At these locations, administration of a HSD was unable to increase the percentages of IL-17A producing cells (Figure 1B). Although IL-17A is considered a signature cytokine of Th17 cells, IL-17A is also secreted by γδ T cells [22], natural killer T (NKT) cells [23], and mucosa-associated invariant T (MAIT) cells, which can be characterized as TCRβ^+^CD4^−^CD8^−/low^CD44^+^ cells in mice [24]. As showed in Figure 1C, we found that 80%-90% of the intestinal IL-17A^+^ cells were CD4^+^TCRβ^+^IL-17A^+^ cells (Th17 cells), which is consistent with a previous report [12]. Furthermore, of the IL-17A producing cells, HSD improved the percentage of CD4^+^TCRβ^+^ cells but decreased the percentage of γδ T cell (Figure 1C). Compared with CD4^+^TCRβ^+^ cells, the fraction of γδ T, MAIT and NKT cells was very low in the IL-17A^+^ intestinal cells in both HSD and ND mice (Figure 1C). Compared to mice on a ND, the absolute number of CD4^+^TCRβ^+^ cells was increased in the small intestine following a HSD, but the numbers of γδ T cell, MAIT cell, and NKT cell were comparable between mice on either a ND or HSD (Figure 1D). Since innate lymphoid cells (ILCs) are also a critical source of IL-17A [25], we further analyzed the IL-17A production of ILCs (lineage^−^CD45^+^CD90.2^+^). HSD increased IL-17A production of SI LP ILCs, but did not affect IL-17A production of ILCs from MLN, PP and spleen (Figure 1E-1F). Moreover, HSD did not change the percentage of ILCs from SI LP, MLN, PP and spleen (Figure 1G). Moreover, the cell fraction of the ILCs in SI LP IL-17A^+^ cells was very low and not significantly affected by a HSD (Figure 1H). These results suggest that a HSD selectively increases IL-17A producing cells in the intestinal LP and that these cells are mainly composed of Th17 cells.

### HSD stimulates the intestinal LP Th17 response

**Figure 2:**
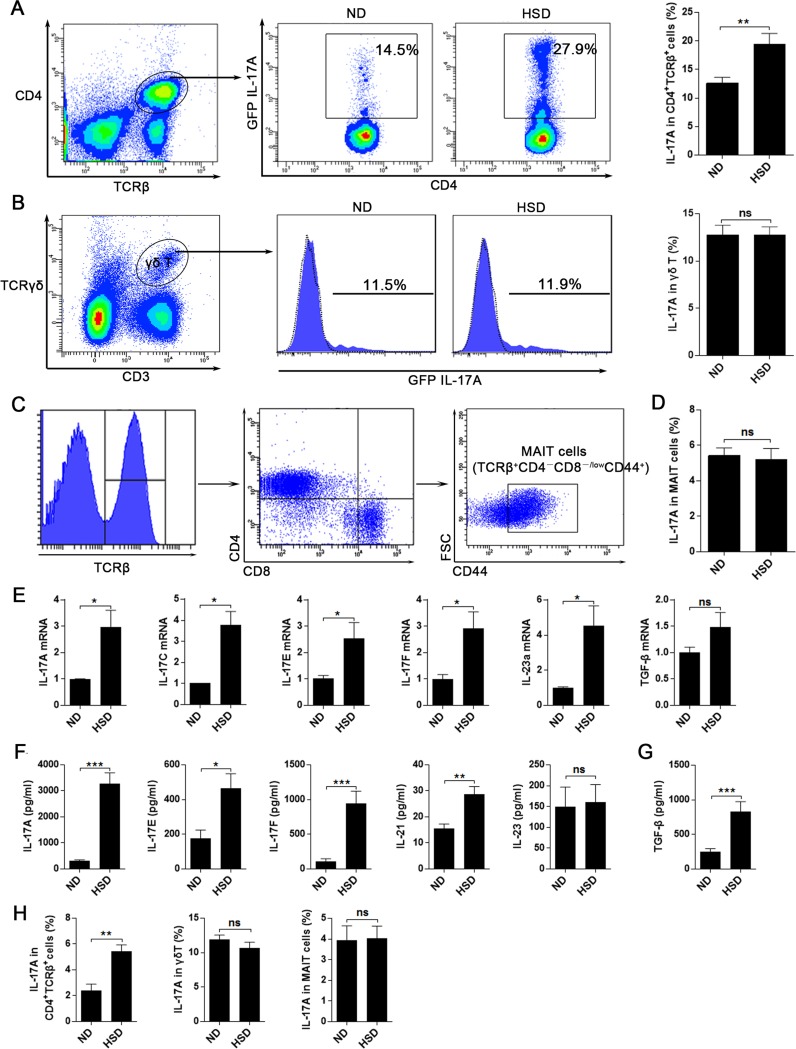
HSD leads to an increased Th17 response in intestinal lamina propria **A.**-**D.** Fresh isolated SI LP lymphocytes from specified diet (3 weeks) mice were incubated for 4 hr with PMA/Ionomycin and GolgiPlug, and intracellular GFP IL-17A expression was evaluated by flow cytometry. A, proportion of IL-17A^+^ cells within the CD4^+^TCRβ^+^ SI LP lymphocytes from specified diet IL-17A-GFP mice (*n* = 8). B, proportion of IL-17A^+^ cells within the SI LP γδ T cells (*n* = 6), filled flow cytometric histograms represent the IL-17A-GFP mice and the dashed line histograms represent the WT mice. C, the flow cytometric gated program of the MAIT cells. D, proportion of IL-17A^+^ cells within the SI LP MAIT cells (*n* = 6). **E.** the mRNA expression of IL-17A, IL-17C, IL-17E, IL-17F, IL-23a and TGF-β, data were acquired from the fresh isolated SI LP cell from the mice exposed to 3 weeks of ND or HSD (*n* = 8). **F.**-**G.** comparison of the IL-17A, IL-17E, IL-17F, IL-21, IL-23 and TGF-β secreting levels of the SI LP cells from the mice exposed to 3 weeks of ND or HSD, fresh isolated SI LP cells were cultured in vitro with the stimulation of PMA and Con A for 12hr, than the supernatant were collected and analyzed (*n* = 8). **H.** fresh isolated LI LP lymphocytes from specified diet (3 weeks) mice were incubated for 4 hr with PMA/Ionomycin and GolgiPlug, and intracellular GFP IL-17A expression of CD4^+^TCRβ^+^ cells, γδ T cells and MAIT cells were evaluated by flow cytometry (*n* = 6). Data are expressed as mean ± SEM from three independent experiments.

While it is known that increased concentration of NaCl enhances the Th17 response *in vitro* and in EAE mice experiments [8], it is still unclear whether HSD alters Th17 response in the gut. In contrast to ND mice, we found the IL-17A production from CD4^+^TCRβ^+^ cells in SI LP was nearly doubled in mice fed a HSD (Figure 2A). However, there was no difference in IL-17A secretions of SI LP γδ T (Figure 2B) and MAIT cells (Figure 2C-2D) between the two groups. To further analyze the effects of HSD on the intestinal LP Th17 response, we measured other Th17 related members of the SI LP lymphocytes. We found that HSD increased the mRNA expression of IL-17A, IL-17C, IL-17E, IL-17F and IL-23 (Figure 2E). Moreover, *in vitro* experiments using SI LP cells demonstrated that HSD increases the secretion of cytokines involved in Th17 response, including IL-17A, IL-17E, IL-17F, IL-21 and TGF-β (Figure 2F-2G). We also found that gene and protein expression of IL-17A, IL-17F and IL-21were significantly increased in purified CD4^+^TCRβ^+^ cells of HSD SI LP lymphocytes when compared to those levels observed in ND controls (Supplementary Figure 3). These data suggest that HSD increase the Th17 response of the small intestine LP.

In the large intestine, the IL-17A secretion level of CD4^+^TCRβ^+^ cells in the LI LP was nearly tripled in HSD mice as compared to ND mice (Figure 2H). It should be noted that HSD did not increase the IL-17A producing level of LI LP γδ T and MAIT cells (Figure 2H). Collectively, our data indicates that HSD enhanced the Th17 response in both the small and large intestine. Besides, we found the stimulatory effect of HSD on IL-17A production of CD4^+^TCRβ^+^ cells was not seen in SI IEL, MLN, PP or the spleen (Supplementary Figure 4), suggesting that HSD selectively promotes the Th17 response in the intestinal LP.

### HSD does not alter T helper 1 (Th1) response but impairs the Th17:Treg balance

**Figure 3:**
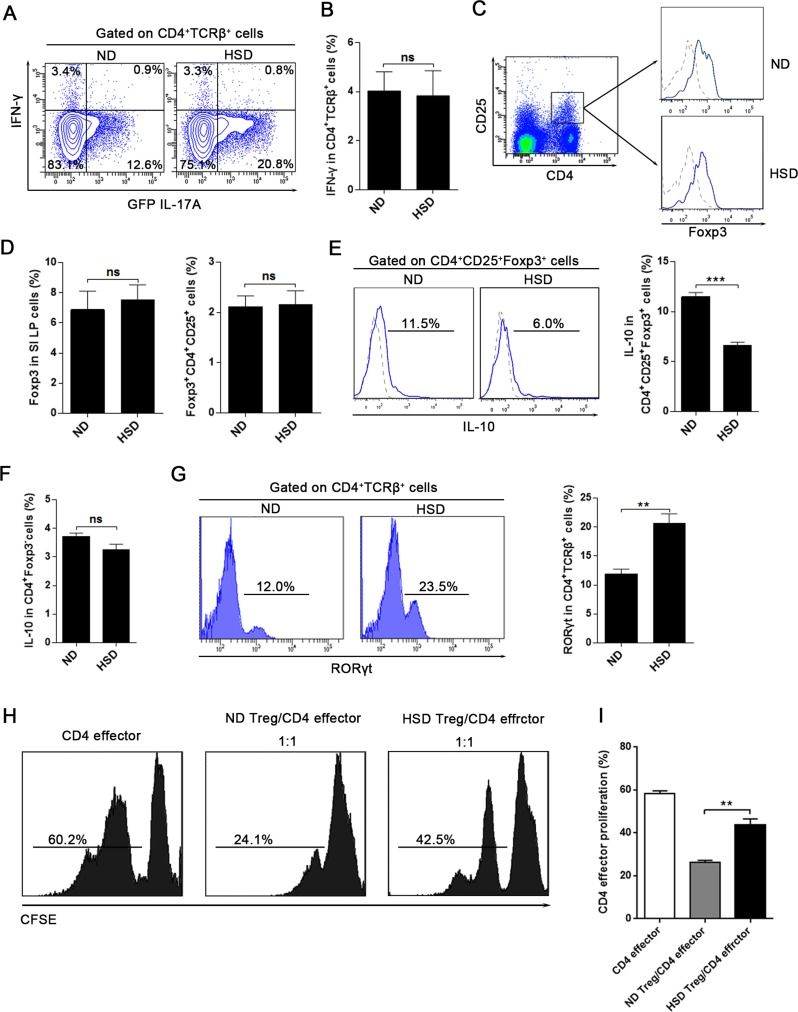
HSD does not alter T helper 1 (Th1) response but impairs the Th17:Treg balance **A.** and **B.** IL-17A-GFP mice were exposed to 3 weeks of ND or HSD before sacrifice, the proportion of IFN-γ^+^ and GFP IL-17A^+^ cells within the CD4^+^TCRβ^+^ SI LP lymphocytes were analyzed (*n* = 6), data were acquired by intracellular staining from the SI LP lymphocytes which were incubated for 4 hr with PMA/Ionomycin and GolgiPlug. **C.**-**G.** Measurements of the SI LP cells that isolated from the mice on a ND or HSD diet for 3 weeks. C, the flow cytometric gating strategy for CD4^+^CD25^+^Foxp3^+^ Treg cells, the Foxp3 level was analyzed after gated on CD4^+^CD25^+^ cells, in the right panel, dash line represent the Foxp3 isotype control and the solid line represent the Foxp3 staining. D, the summative data of the Foxp3 expression and the percentages of CD4^+^CD25^+^Foxp3^+^ Treg cells in the SI LP cells (*n* = 8). E-F, the IL-10 production of the CD4^+^CD25^+^Foxp3^+^ cells and the CD4^+^Foxp3^−^ cells, fresh isolated SI LP cells were incubated for 4 hr with PMA/Ionomycin and GolgiPlug before intracellular staining by True-Nuclear^TM^ Transcription Factor Buffer Set. G, the RORγt level of the SI LP CD4^+^TCRβ^+^ cells (*n* = 6). H-I, Treg suppression assay, the CD4 effector cells (CD4^+^CD62L^+^CD44^−^) were sorted from mice exposed to 3 weeks of ND, the CD4^+^CD25^+^ Treg were sorted from mice exposed to 3 weeks of ND or HSD, CD4 effector cells were labeled with CFSE, stimulated with CD3/CD28 Dynabeads and cultured alone or co-cultured with Treg at ratios as indicated (with IL-2). **H.** CFSE dilution of CD4 effector cells was measured by flow cytometry after 3.5 days, and CFSE dilution was obtained after gating on CD62L^+^ cells (CD4 effector cells). **I.** Summary of the proliferation rate of the CD4 effector cells. Data are expressed as mean ± SEM from three independent experiments.

Th1 cells are another subset of CD4^+^ T cells and IFN-γ is their major effector cytokine. We found that a HSD simulates the production of IL-17A in CD4^+^TCRβ^+^ cells, but does not increase the expression of IFN-γ (Figure 3A-3B). Similarly, the expression of IFN-γ was not influenced in NK or CD8 T cells in mice fed a HSD (Supplementary Figure 5). The intestinal LP contains large numbers of Treg cells, and Th17:Treg balance is crucial in maintaining a normal intestinal immunity [12]. We found that a HSD had no significant effect on the percentage of Treg cells (CD4^+^CD25^+^Foxp3^+^) of the SI LP (Figure 3C-3D) and the LI LP (Supplementary Figure 6A). Additionally, HSD did not alter the expression of Foxp3 in the SI LP and LI LP (Figure 3D and Supplementary Figure 6A). However, we found that the IL-10 production of the SI LP and LI LP CD4^+^CD25^+^Foxp3^+^ cells was significantly decreased in the HSD mice compared to the ND counterparts (Figure 3E and Supplementary Figure 6B). HSD did not inhibit the IL-10 expression of CD4^+^Foxp3^−^ cells (Figure 3F and Supplementary Figure 6B). More importantly, using an *in vitro* co-culture suppression assay system, we found the suppressive function of the Treg cells from HSD mice was significantly decreased compared to ND controls (Figure 3H-3I). Thus, although HSD did not alter the percentage of Treg cells, HSD inhibits the suppressive function of Treg cells. Previous study have reported that excess NaCl inhibits the suppressive function of Treg but increases IFN-γ expression [26]. In keeping with these findings, we similarly found that HSD increase the IFN-γ level of the SI LP Treg cells (Supplementary Figure 6C-6D). We examined the expression of RORγt and found that RORγt was significantly increased in the SI LP CD4^+^TCRβ^+^ cells of HSD mice compared to the ND (Figure 3G). Together, these data indicate that a HSD disrupts the Th17:Treg balance.

### HSD aggravates the TNBS-induced mice colitis

**Figure 4:**
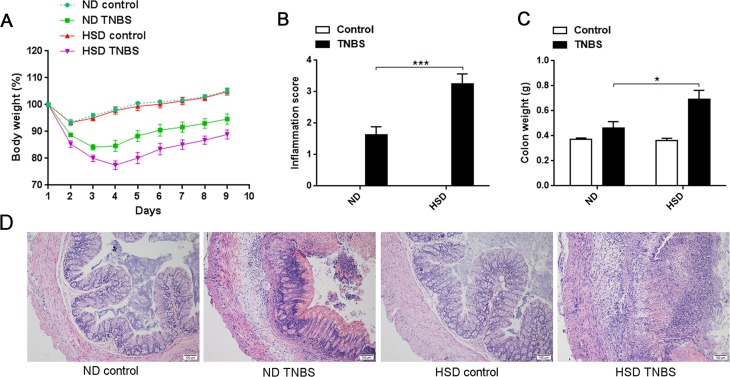
Actue TNBS-induced colitis was exacerbated in mice fed a HSD **A.** Weight loss of mice after induction of TNBS colitis, IL-17A-GFP mice were subjected to a ND or HSD before (3 weeks) and after TNBS or ethanol treatment, and the mice were weighed daily after colitis induced (*n* = 8). **B.** Colon inflammation was graded on the basis of the criteria as described, and data was acquired 3 days after colitis induction (*n* = 6). **C.** The mice colon weight was acquired 3 days after colitis induction (*n* = 6). **D.** Representative H&E-stained colon cross-sections 3 days after colitis induction, scale bar = 100μm. Data are expressed as mean ± SEM from three independent experiments.

Previous studies have reported that Th17 cells and Th17 associated molecule may play a critical role in the pathogenesis of IBD [15–17]. To address whether HSD increases the susceptibility of mice to colitis, both acute and chronic TNBS-induced mice colitis model were established. As showed in Figure 4A, the weight loss of acute TNBS-induced colitis was more severe in the HSD mice compared to ND. We also measured the colonic histological inflammation score based on the severity of hemorrhage, hyperemia, and edema. This analysis also indicated an aggravated TNBS-induced colitis in the HSD mice (Figure 4B). Moreover, we analyzed the weight of the colon and found this was significantly increased in the TNBS-treated mice fed a HSD (Figure 4C). The colons from ND and HSD control mice displayed normal histology while TNBS-treated mice displayed conspicuous colonic inflammation (Figure 4D). Importantly, when compared to the ND TNBS-treated mice, the colons of HSD TNBS-treated mice demonstrated extensive leukocyte infiltration, severe destruction of villi and edema of the colonic wall (Figure 4D).

In the chronic TNBS colitis experiments, we found colitis was more severe in HSD TNBS group compared to the ND TNBS group (Supplementary Figure 7B-C). Compared to the ND TNBS group, the collagen expression in the colon was increased in the HSD TNBS group as shown by MT staining and collagen RT-PCR (Supplementary Figure 7B and 7D). Moreover, the survival rate was significantly decreased in the HSD TNBS group compared to the ND TNBS group (Supplementary Figure 7C). These data confirm that HSD aggravates the TNBS-induced mice colitis, and further suggest HSD may be an important risk factor for IBD.

### HSD stimulates a Th17 response in TNBS-induced mice colitis

**Figure 5:**
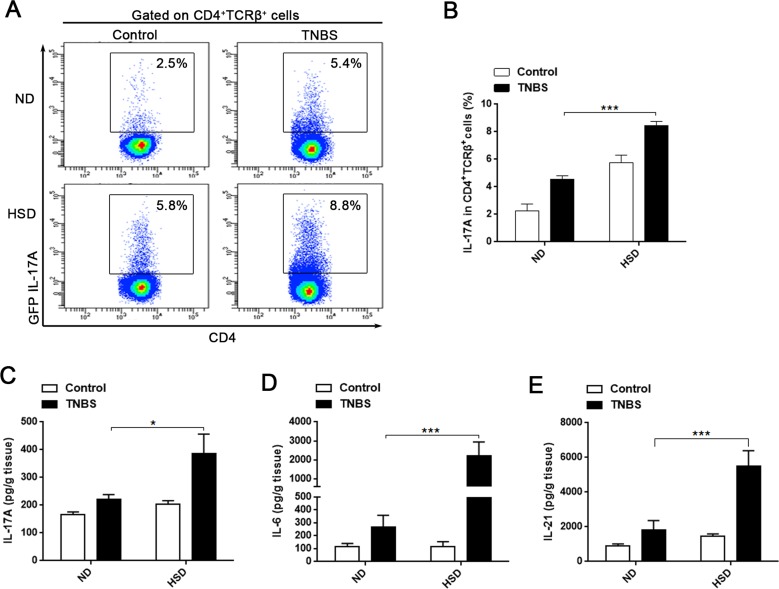
Th17 response was stimulated in acute TNBS-induced colitis of mice fed a HSD **A.** and **B.** the proportion of GFP IL-17A^+^ cells within the CD4^+^TCRβ^+^ LI LP lymphocytes. Cells were isolated from specified diet IL-17A-GFP mice 3 days after colitis induction and incubated for 4 hr with PMA/Ionomycin and GolgiPlug (*n* = 8). **C., D.** and **E.** ELISA analysis of the colonic IL-17A (C), IL-6 (D) and IL-21 (E) of ND or HSD colitis-induced mice, colonic tissues were sampled from specified diet mice 3 days following induction of colitis (*n* = 8). Data are expressed as mean ± SEM from three independent experiments.

In the present study, we demonstrated that the intestinal LP Th17 response is enhanced in mice fed a HSD, and HSD aggravates TNBS-induced colitis. Since previous studies have confirmed a critical role of Th17 in pathogenesis of IBD [15–17], we speculated that HSD may aggravate TNBS-induced colitis through increased Th17 response. To evaluate this hypothesis, we analyzed the colonic Th17 response in ND or HSD TNBS-treated mice. Compared to the ND or HSD control mice, the IL-17A production of LI LP CD4^+^TCRβ^+^ cells was significantly increased in the TNBS-treated ND or HSD mice, respectively (Figure 5A-5B). Among the TNBS-treated mice, the IL-17A producing level was much higher in the HSD mice compared to the ND (Figure 5A-5B). In addition, we measured the colonic IL-17A, IL-6 and IL-21 level by ELISA. We found these cytokines were increased in the HSD control mice compared to the ND counterparts, and these cytokines was much higher in the TNBS-treated HSD mice as compared to the TNBS-treated ND mice (Figure 5C-5E). In the chronic TNBS induced colitis model, we found the colonic IL-17A was significantly increased in HSD TNBS treated mice as compared to the ND TNBS treated mice (Supplementary Figure 7E). In addition, in the chronic TNBS induced colitis, colonic IL-6, TGF-β, and IFN-γ were also significantly increased in the TNBS-treated HSD mice, but IL-10 was decreased in the TNBS-treated HSD mice (Supplementary Figure 8). These data indicate that HSD aggravates TNBS-induced mice colitis is related to the enhanced Th17 response, and may also be related to the impaired Treg suppression. Furthermore, we performed *in vitro* experiment to analyze the relationship between Th17 response and colitis. The Caco-2 cell line is derived from intestinal epithelial cells and is often used in the research of IBD [27]. A cytotoxic assay demonstrated that IL-17A significantly increased the cytotoxicity of PBMC against Caco-2 cells (Supplementary Figure 9), and suggested that IL-17A may aggravates the colonic tissue injury through increasing the cytotoxicity of lymphocytes against the intestinal epithelial cell.

## DISCUSSION

In the last three to four decades, the incidence of autoimmune diseases such as multiple sclerosis (MS), type 1 diabetes, and IBD have been steadily increasing [28]. Simultaneously, there has been widespread adoption of diets dependent on processed foods rich in salt, fats, and sugar as well as artificial sweeteners [28]. Th17 cells is crucial in the pathogenesis of autoimmune diseases, and studies found that excess of NaCl stimulates Th17 response and worsens the severity of autoimmune diseases [7, 8]. However, the effect of HSD on the lower gastrointestinal tract Th17 response and the association between HSD and IBD remain poorly characterized. In the present study, we demonstrated that HSD selectively promotes the accumulation of IL-17A producing cells and increases the Th17 response of the intestinal LP, and HSD impairs the function of Treg cells. We also found that a HSD exacerbates the inflammatory response in TNBS-induced colitis, which suggesting HSD is a risk factor for IBD.

The detrimental effects of a HSD on human health have received much attention in the past, and the typical hazard attributed to a HSD is related to its effects on blood pressure and cardiovascular disease [29]. Recently, there is growing evidence that excessive NaCl uptake alters the immune system in both mice and humans [7–10]. And two independent studies have shown that increased salt intake can lead to a markedly enhanced Th17 response and increase the severity of an autoimmune disease (EAE) [7, 8].

In this present study, we demonstrated that the percentage of IL-17A producing cells of ND mice were significantly higher in the intestinal LP than in other lymphoid sites, including the SI IEL, CI IEL, MLN, and PP. The proportion of CD4^+^TCRβ^+^ that expressed IL-17A was about 10%-15% in the SI LP and 2%-3% in the LI LP in mice fed a normal diet. Importantly, we found that high salt intake increases the percentage of IL-17A producing cells of the lamina propria lymphocyte, and 80%-90% IL-17A producing cells are CD4^+^TCRβ^+^IL-17A^+^ cells (Th17 cells). γδ T cell, MAIT cell and ILC cells are relatively small population compared to Th17 cells. Moreover, of IL-17A producing cells, the fraction of Th17 cells is increased in the HSD-fed mice. Our data revealed that the mRNA expression of IL-17A, IL-17C, IL-17E, IL-17F and IL-23 are increased in the SI LP cells from the HSD-fed mice, the SI LP cells from HSD-fed mice acquired stronger production for IL-17A, IL-17E, IL-17F IL-21 and TGF-β. Different from other Th17 associated cytokines, IL-17E was reported negatively regulate the Th17 response [30]. Therefore, while the specific effect of HSD on IL-17E requires further investigation, we speculate that it may be due to a feedback effect to the enhanced Th17 response. Notably, the HSD effect on intestinal LP lymphocytes is selectively, HSD did not alter the IFN-γ production levels of CD4^+^TCRβ^+^ cell, NK cell and CD8 T cells.

Importantly, we found high salt intake impaired the gut Th17:Treg balance. RORγt is a critical transcription factors that drive CD4^+^ T cell develop into Th17 cells, we found HSD significantly increases RORγt expression of the SI LP CD4^+^TCRβ^+^ cells. Although the Foxp3 level and the percentage of CD4^+^CD25^+^Foxp3^+^ Treg population were not altered by HSD, the IL-10 production of Treg cells was sharply decreased in the HSD mice as compared to ND mice. We demonstrated that the suppressive function of the Treg cells from HSD mice was significantly decreased compared to the ND mice. Our results are consistent with recent reports by Hernandez *et al.* [26], that found increasing NaCl markedly impairs suppressive function of Treg. Collectively, our data demonstrates that a HSD increased intestinal Th17 response but inhibited Treg suppressive function in mice. Thus, HSD might increase the risk of intestinal autoimmune disease by impairing the gut Th17:Treg balance.

Th17 is a subset of CD4^+^ T cells with IL-17A as major cytokine, and TGF-β, IL-6, and IL-21 are cytokines that promote the differentiation of naive T cells into mature Th17 cells [11]. In the current study, we demonstrate that colonic IL-17A, IL-6 and IL-21 are significantly increased in TNBS-treated mice that fed a HSD compared to ND mice. This finding suggests that HSD aggravates colitis in mice, potentially through increases the Th17 response and promotes differentiation of the CD4^+^ T cell into Th17 cells. We further demonstrated that IL-17A may aggravate the colonic tissue injury by increasing the cytotoxicity of lymphocytes against the intestinal epithelial cell. Interestingly, a previous study reported that commensal microbes and segmented filamentous bacterium (SFB) were capable of inducing the appearance of intestinal LP Th17 cells [31]. Therefore, HSD may affect the intestinal Th17 cells by altering the intestinal microflora, or by increasing the concentration of NaCl. However, the exact mechanism by which HSD increases intestinal Th17 response requires further investigation.

The incidence of IBD is increasing, not only in Europe and North America, but also in the areas with known high IBD-related morbidity [21]. Poor eating habits and the spread of the Western diet have been considered as a possible explanation for the increase incidence of IBD [21, 32]. It has been reported that there is a positive association between high dietary intakes of total fats, total polyunsaturated fatty acids, omega-6 fatty acids, and meat and increased IBD risk [21]. However, there is a negative association between high fiber, fruit and vegetable intake and IBD risk [21]. High NaCl intake is a characteristic of the Western diet, but it is still unclear the degree to which a HSD influences IBD incidence. In this study, we found high dietary intake of NaCl was a risk factor for IBD incidence in an animal model, suggesting that HSD may be a contributing factor in this disease. And a recently published study also suggested that HSD contribute to exacerbation of IBD, they found that HSD aggravate TNBS and dextran sulfate sodium (DSS) induced colitis in Balb/c mice [33].

In conclusion, our findings demonstrate that high dietary intake of NaCl changes intestinal immunity, enhances the Th17 response and disturbs the Th17:Treg balance. HSD significantly increase the severity of TNBS-induced mice colitis, and this is related to the enhanced Th17 response. The results of this study suggest that the increased adoption of the Western diet increase the risk of IBD, and reduced dietary intake of NaCl may be helpful decrease the rate of IBD relapse, alleviate IBD symptoms, or even reduce the risk of developing IBD.

## MATERIALS AND METHODS

### Animals

IL-17A-GFP reporter mice, with a C57BL/6 genetic background, were purchased from Biocytogen (Worcester, MA, USA). Wild-type (WT) C57BL/6 mice were purchased from Shanghai SLAC Laboratory Animal Company. Six-week-old male mice were randomized into control and experimental groups. All experimental procedures were approved by the Animal Care Ethics Committee of the First Affiliated Hospital, Zhejiang University (Permit number: 2015-187), and all animals received humane care according to the “Guide for the Care and Use of Laboratory Animals” published by the National Institutes of Health.

### High salt diet and acute TNBS-induced colitis induction

Mice received normal chow and water ad libitum (ND) or a sodium-rich chow containing 4% NaCl and water containing 1% NaCl ad libitum (HSD) [8]. Mice were fed on specified diet for 3 weeks and then either sacrificed or treated with TNBS to induce colitis. In brief, mice were anesthetized by intraperitoneal injection of chloral hydrate (0.4 g/kg), a blunt-tip steel gavage needle was inserted into the rectum of mice, then 2.5 mg of TNBS in 50% ethanol (vol/vol) or vehicle (50% ethanol) alone was introduced into the colon through a needle inserted 4 cm into the anal canal. Following induction, mice were kept on their respective diet (ND or HSD). Three days after the induction of colitis, mice were sacrificed to evaluate intestinal inflammation, as previously described [34]. Macroscopic signs of inflammation were scored and documented, as previously described [16], according to the following scheme: 0 = no visible edema or hemorrhage; 1 = focal hyperemia or bowel wall edema and no hemorrhage; 2 = focal hyperemia or bowel wall edema and hemorrhage at 1 site; 3 = extended hyperemia or bowel wall edema and hemorrhage at >1 site; and 4 = extended hyperemia or bowel wall edema, hemorrhage at >1 site, and perforation [16].

### Chronic TNBS colitis induction

Chronic TNBS-induced colitis was induced using four times of TNBS in 55% ethanol. Control mice were given the vehicle (55% ethanol) only. The doses of TNBS were 2.0mg, 2.5mg, 2.5mg and 2.5mg. After the first administration of TNBS (day 0), three additional doses were given at day 7, day 11, and day 14. The mice were sacrifice on day 17, after initial administration of TNBS solution or ethanol. Mice were kept in their specified diet during the induction of chronic TNBS colitis.

### Cell isolation and flow cytometry

Intraepithelial lymphocytes (IEL) and LP lymphocytes were isolated with only minor modification to previously described methods [14]. The residual mesenteric fat tissue and Peyer's patches (PP) were removed, and the intestine was opened longitudinally. After thoroughly washed in ice-cold PBS, the intestine was cut into 1.0 cm pieces. To obtain IEL, intestine pieces were shaken twice at 250 rpm for 20 min at 37°C in HBSS medium supplemented with 5% FBS (Gibco) containing 2 mM EDTA. To obtain LP lymphocytes, the remaining intestinal tissue from EDTA digestion was washed, minced and shaken once at 250 rpm for 25 min at 37°C in HBSS with 5% FBS containing type VIII collagenase at 1.5 mg/ml (Sigma-Aldrich) [35]. Supernatants from EDTA or collagenase digestion were collected, washed once in cold PBS and resuspended in Percoll. After Percoll gradient separation [14], the (IEL) and LP lymphocytes were collected at the interphase of the Percoll gradient. The purity analysis showed that the isolated IEL have very high purity (Supplementary Figure 1). Splenocytes were prepared by grinding splenic fragments through a mesh filter, which was then treated with a lysis solution to remove intact RBCs. Cells from mesenteric lymph node (MLN) and PP were prepared as previously described [36]. For intracellular staining, cells were incubated for 4 hr with PMA (50 ng/ml) and ionomycin (750 ng/ml; both from Sigma-Aldrich) in the presence of GolgiPlug (BD Biosciences) at 37°C. Because we used IL-17A-GFP reporter mice in this study, intracellular staining for IL-17A was not required, and a more detailed protocol of surface and intracellular staining has been previously described [37, 38]. For staining of transcription factors, True-Nuclear^TM^ Transcription Factor Buffer Set (Biolegend) was used according to the manufacturer's protocol. The antibodies used in this study including anti-CD4-PE (GK1.5), anti-CD4-BV510 (RM4-5), anti-CD8-BV421 (53-6.7), anti-NK1.1-PE (PK136), anti-CD25-APC (PC61) (all from BD Biosciences), anti-CD3-FITC (17A2), anti-TCRγδ-APC (GL3), anti-CD103-Alexa Fluor^®^ 488 (2E7), anti-TCRγδ-APC (GL3), anti-IFN-γ-APC (XMG1.2), lineage cocktail-PE (Biolegend, Cat.133303, PE anti-mCD3ε/PE anti-mGr-1/PE anti-mCD11b/PE anti-mCD45R(B220)/PE anti-mTer-119), lineage isotype control cocktail-PE (Biolegend, Cat.133303), anti-CD45-Pacific Blue (30-F11), anti-CD90.2-APC (30-H12), anti-CD4-PerCP/Cy5.5 (GK1.5), anti-FOXP3-PE (MF-14), PE IgG2b,κ Isotype control (RTK4530), anti-IL10-Brilliant Violet 421^TM^ (JES5-16E3), anti-CD8a-PE (53-6.7), anti-CD62L-APC (MEL-14), anti-CD44-PerCP/cy5.5 (IM7) (all from Biolegend), and anti-TCRβ-PE (H57-597), anti-TCRβ-APC (H57-597), anti-RORγt-APC (AFKJS-9) (all from eBioscience). Data were acquired on a FACSCanto™ II flow cytometer (BD Biosciences) and analyzed with FlowJo (Treestar) or BD FACS Diva (BD Biosciences) software.

### Histological analyses

To analyze morphological changes, colon samples were paraffin-embedded, sectioned and stained with hematoxylin and eosin (H&E) or Masson trichrome (MT).

### Cell culture and Treg suppression assay

Cells were cultured in RPMI Media 1640 (Gibco) supplemented with 10% FBS (Gbico), 100 U/ml penicillin and 100 mg/ml streptomycin. In analysis of the cytokine secretion ability, 2.0×10^6^/well LP lymphocytes in 1ml culture medium were cultured in 24-well plates, and stimulated with PMA (50 ng/ml) and Concanavalin A (Con A,10μg/ml) for 12-hours. The supernatant was collected for cytokine analysis.

To analyze the suppressive function of regulatory T cells (Treg), CD4^+^ naive effector T cells (CD4 effector cells, CD4^+^CD62L^+^CD44^−^) and Treg cells (CD4^+^CD25^+^) were sorted from the mice splenocytes by a FACS Aria III instrument (Becton Dickinson), the purity of the sorted cells was > 95% as verified by post-sort analysis. CD4^+^ naive effector T cells (2 × 10^5^) were stained with Cell Trace CFSE and cultured with Treg (2 × 10^5^) in 96-well plate. Cells were stimulated with Mouse T Activator CD3/CD28 Dynabeads (life technologies, 2 beads/CD4 effector cells) in the presence of IL-2 (10 U/ml) and culture for 3.5 days before FACS analysis.

### Cytokine analysis

IL-17A, IL-6, IL-21 and TGF-β levels were measured using mouse enzyme-linked immunosorbent assay (ELISA) Ready-Set-Go Kits (eBioscience) as specified by the manufacturer. Cytokine contents of intestinal extracts are expressed as amount per gram of tissue. Th17-associated cytokines, including IL-17A, IL-17E, IL-17F, IL-23 and IL-21, were assessed using the MILLIPLEX^®^ MAP Mouse Th17 Magnetic Bead Panel on Luminex200 according to the manufacturer's protocol.

### Analysis of mRNA expression

Total RNA was isolated using Trizol (Takara), and cDNA was synthesized using a PrimeScript^TM^ RT Master Mix (Takara) according to the manufacturers' instructions. Gene expression was quantified using the comparative CT method, with CT values normalized to β-actin. PCR was performed using SYBR^®^ Premix Ex Taq^TM^ II (Takara) with specific primers as follows:

*IL23a*: 5′-AATAATGTGCCCCGTATCCAGT-3′ (sense), 5′-GCTCCCCTTTGAAGATGTCAG-3′ (anti—sense).

*TGFβ*: 5′-CTCCCGTGGCTTCTAGTGC-3′ (sense),

5′-GCCTTAGTTTGGACAGGATCTG-3′ (anti-sense).

*IL17F*: 5′-TGCTACTGTTGATGTTGGGAC-3′ (sense),

5′-AATGCCCTGGTTTTGGTTGAA-3′ (anti-sense).

*IL-17E*: 5′-ACAGGGACTTGAATCGGGTC-3′ (sense),

5′-TGGTAAAGTGGGACGGAGTTG-3′ (anti-sense).

*IL17C*: 5′-TCTGCTGAGGAATTATCTCACGG-3′ (sense),

5′-GTTCCAGCTAGAGGTCCTTCA -3′ (anti-sense).

*IL17A*: 5′-TTTAACTCCCTTGGCGCAAAA-3′ (sense),

5′-CTTTCCCTCCGCATTGACAC-3′ (anti-sense).

*Collagen*: 5′-CAACAGTCGCTTCACCTACAG-3′ (sense),

5′-GGAGGTCTTGGTGGTTTTGTAT-3′ (anti-sense).

*β-actin*: 5′-GAAGATCAAGATCATTGCTCCT-3′ (sense),

5′-TGGAAGGTGGACAGTGAG-3′ (anti-sense).

*IL21*: 5′-GGACCCTTGTCTGTCTGGTAG3′ (sense)

5′-TGTGGAGCTGATAGAAGTTCAGG-3′(anti-sense).

### Statistical analysis

Data are expressed as mean ± SEM and statistical analyses were performed using GraphPad Prism (GraphPad Software). Differences between two groups were analyzed using Student's *t*-test (unpaired, two-tailed), and multigroup comparisons were performed by One-way ANOVA. P<0.05 was considered significant. *P < 0.05, **P < 0.01, ***P < 0.001, ns represents not significant.

## SUPPLEMENTARY MATERIAL


