# Supplementary material for: High salt diet stimulates gut Th17 response and exacerbates TNBS-induced colitis in mice
Source: Oncotarget. 2016 Dec 1;8(1):70–82. doi: 10.18632/oncotarget.13783 (PMC5352190; doi:10.18632/oncotarget.13783)
Supplement: Supplementary file 1 [file oncotarget-08-70-s001.pdf]

High salt diet stimulates gut Th17 response and exacerbates TNBS-induced colitis in mice

Supplementary Material

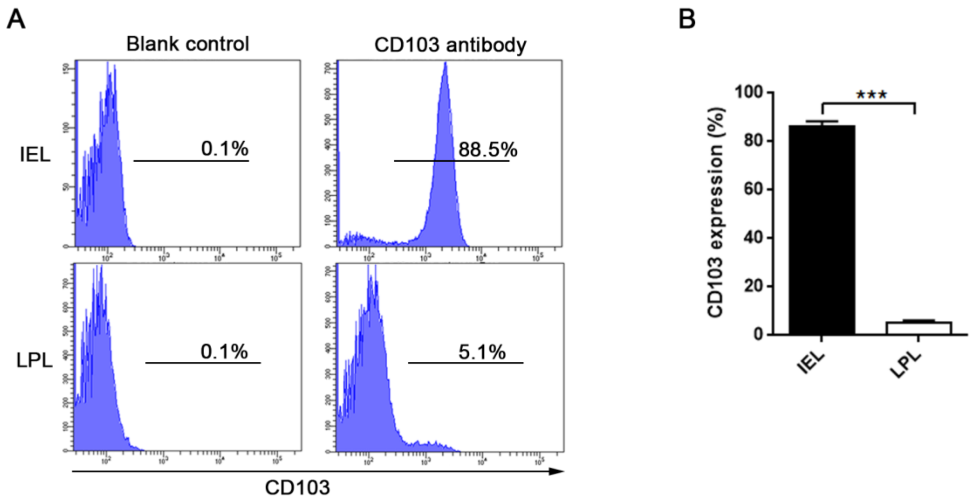

Supplementary Figure 1: Analysis the purity of the intestinal intraepithelial cells by flow cytometry. Intraepithelial lymphocyte (IEL) and lamina propria lymphocyte (LPL) were isolated from the small intestine of the mice with a normal diet, cells were stained with anti-CD103 antibody. A, the CD103 levels of IEL and LPL were measured by flow cytometry. B, the summary of CD103 expression on IEL and LPL, n=6. Data are expressed as mean ± SEM from two independent experiments.

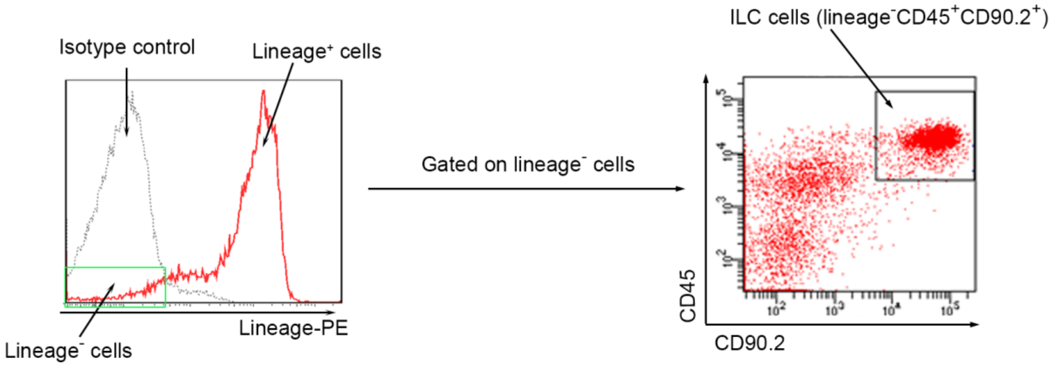

Supplementary Figure 2: The flow cytometric gated program of the ILC cells. ILC cell population was lineage$^-$CD45$^+$CD90.2$^+$, we first gated the lineage$^-$ cells, then gated the CD45$^+$CD90.2$^+$ cells from the lineage$^-$ cells.

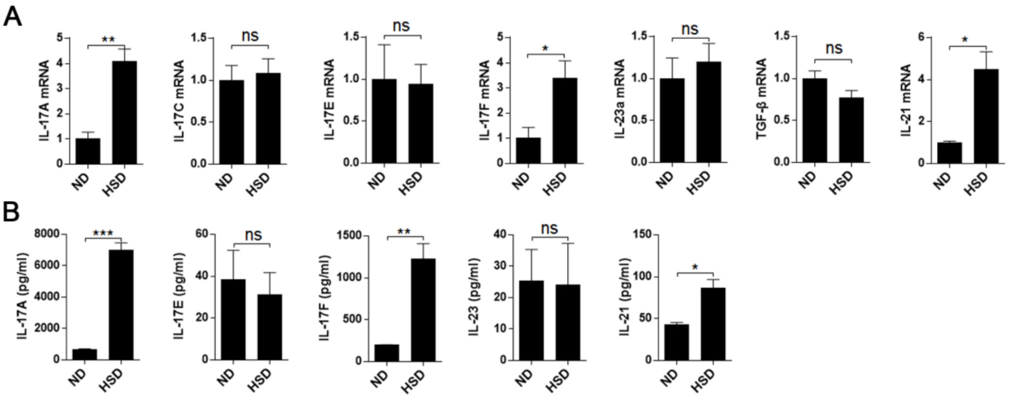

Supplementary Figure 3: HSD increase the Th17 response in purified CD4 T cells. CD4 T cells ($CD4^+TCR\beta^+$) were sorted by FACS Aria III instrument from the fresh isolated SI LP cells of the mice exposed to 3 weeks of ND or HSD. **A**, the mRNA expression of IL-17A, IL-17C, IL-17E, IL-17F, IL-23a, TGF-β and IL-21, data were acquired from the sorted CD4 T cells (n=3). **B**, analysis of the IL-17A, IL-17E, IL-17F, IL-23 and IL-21 secreting levels, sorted CD4 T cells were cultured with the stimulation of PMA and Con A for 12hr, than the supernatant were collected and analyzed by the MILLIPLEX® MAP Mouse Th17 Magnetic Bead Panel on Luminex200 according to the manufacturer's protocol (n=3). Data are expressed as mean ± SEM.

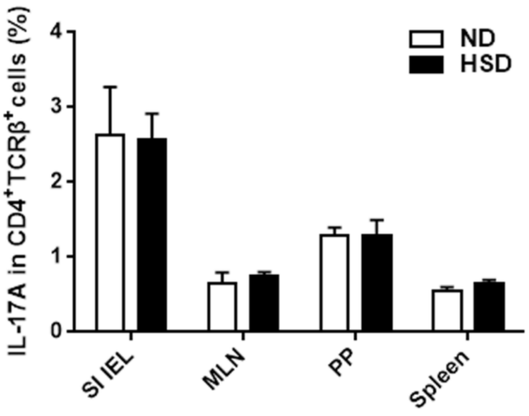

Supplementary Figure 4: HSD selectively stimulates a Th17 cell response. The proportion of GFP IL-17A$^+$ cells within the CD4$^+$TCRβ$^+$ lymphocytes from SI IEL, MLN, PP and spleen. Data was acquired from the IL-17A-GFP mice that were on a HSD or ND for 3 weeks, fresh isolated cells were incubated for 4 hr with PMA/Ionomycin and GolgiPlug before flow cytometric analysis, n=6. Data are expressed as mean ± SEM from three independent experiments.

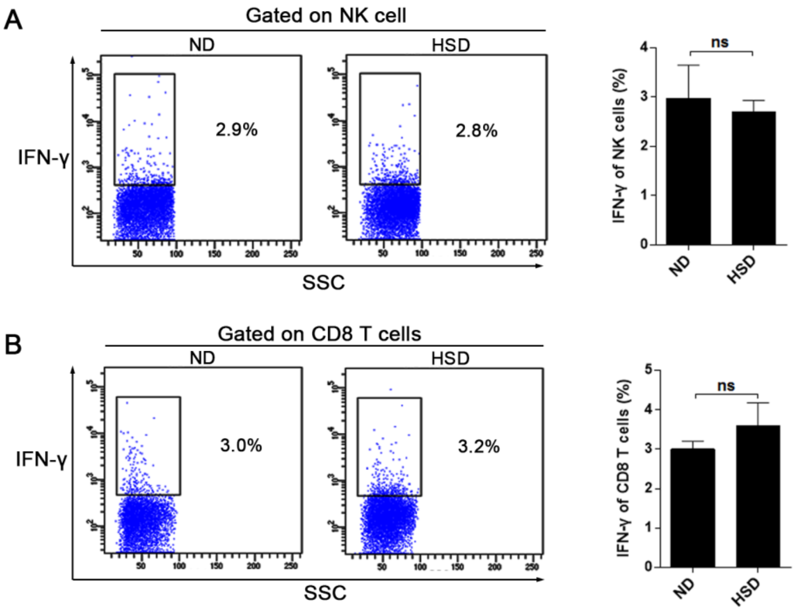

Supplementary Figure 5: IFN-γ production levels of NK and CD8 T cells. Mice were exposed to 3 weeks of ND or HSD before sacrifice, fresh isolated SI LP lymphocytes were incubated for 4 hr with PMA/Ionomycin and GolgiPlug, and then the intracellular IFN-γ expression in **(A)** NK and **(B)** CD8 T cells were examined by flow cytometry, n=4. Data are expressed as mean ± SEM from two independent experiments.

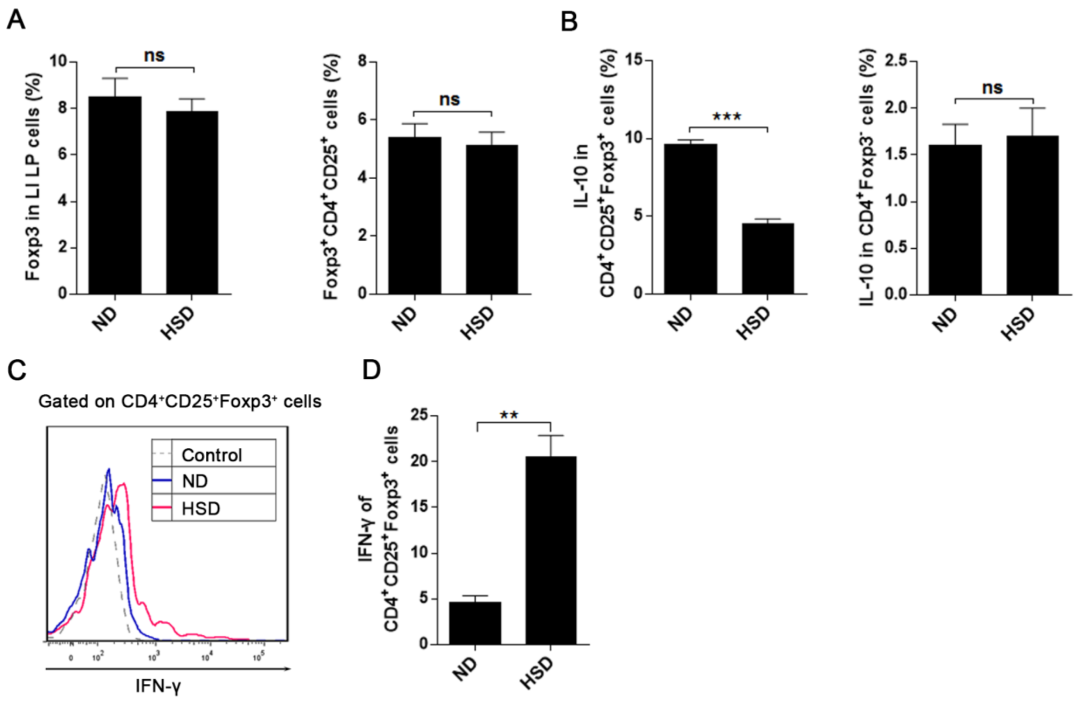

Supplementary Figure 6: Analysis of the Treg cells. Mice were exposed to 3 weeks of ND or HSD before sacrifice. **A**, Measurement of the Foxp3 expression and the percentages of $CD4^{+}CD25^{+}Foxp3^{+}$ Treg in the LI LP cells (n=8), data was acquired by flow cytometry, the gating strategy was showed in Figure 3C. **B**, the IL-10 production of the $CD4^{+}CD25^{+}Foxp3^{+}$ and $CD4^{+}Foxp3^{-}$ cells, fresh isolated LI LP cells were incubated for 4 hr with PMA/Ionomycin and GolgiPlug before intracellular staining by True-Nuclear^{TM} Transcription Factor Buffer Set, n=6. **C-D,** IFN-γ production of the $CD4^{+}CD25^{+}Foxp3^{+}$ Treg cells, data were acquired from the SI LP cells, n=6. Data are expressed as mean ± SEM from three independent experiments.

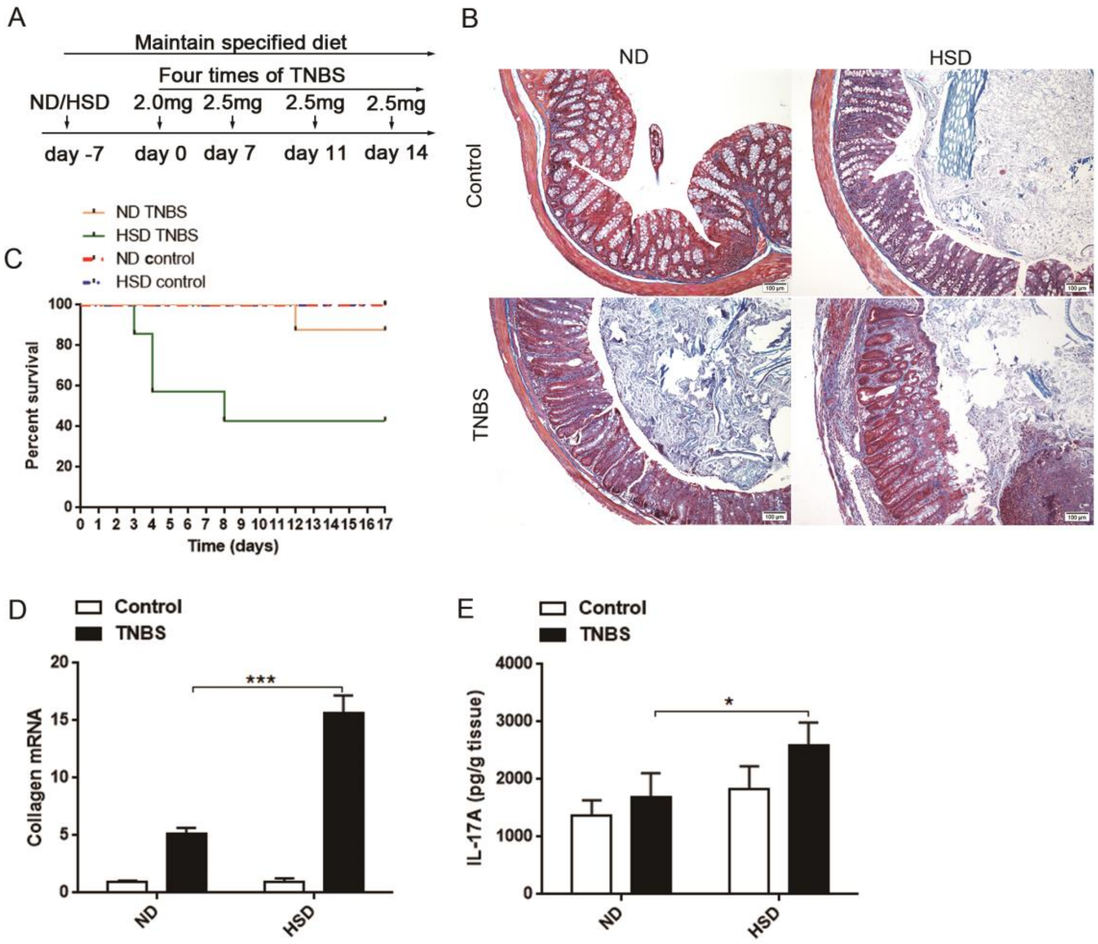

Supplementary Figure 7: TNBS-induced chronic colitis was exacerbated in mice fed a HSD. **A,** the protocol of TNBS induce chronic colitis. **B**，Masson trichrome (MT) staining of representative colon sections on day 17 after initial administration of TNBS solution or ethanol (control), scale bar =100μm. **C,** Survival curve from mice (with ND or HSD) administered with TNBS or ethanol, data was analyzed by the Kaplan–Meier method. **D,** mRNA expression of colon *Collagen* on day 17 after initial administration of TNBS solution or ethanol (control), n=5. **E,** Luminex enzyme immunoassay, by MILLIPLEX® MAP Mouse Th17 Magnetic Bead Panel, analyzed the colonic IL-17A on day 17 after initial administration of TNBS solution or ethanol, n=5. Data are expressed as mean ± SEM from two independent experiments.

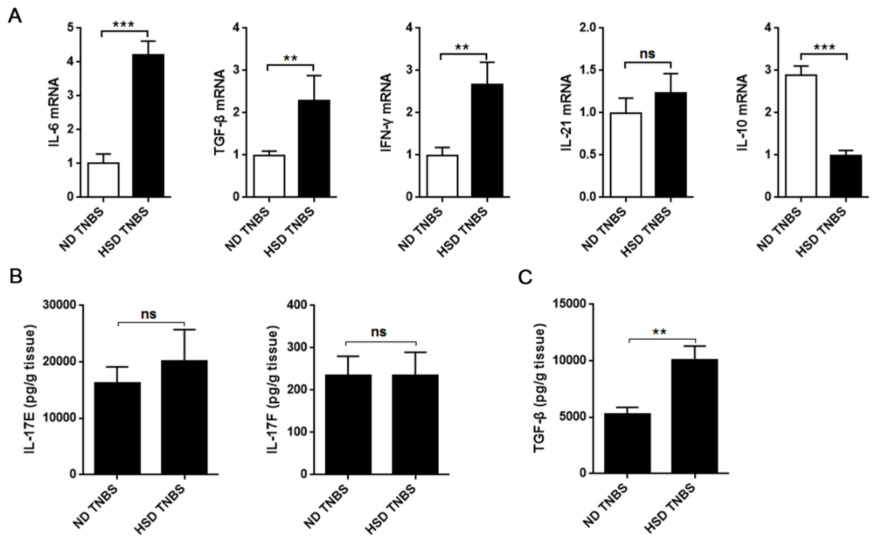

Supplementary Figure 8: Measurement of the colonic cytokines. Colonic tissues were collected from the normal diet (ND) or high salt diet (HSD) mice with TNBS-induced chronic colitis on day 17 after initial administration of TNBS solution. A, IL-6, TGF-β, IFN-γ, IL-21 and IL-10 mRNA of the colonic tissue. B, Luminex enzyme immunoassay, by MILLIPLEX® MAP Mouse Th17 Magnetic Bead Panel, analyzed the colonic IL-17E and IL-17F. C, TGF-β of the colonic tissue was measured by enzyme-linked immunosorbent assay (ELISA). Data are expressed as mean ± SEM from two independent experiments, n=6.

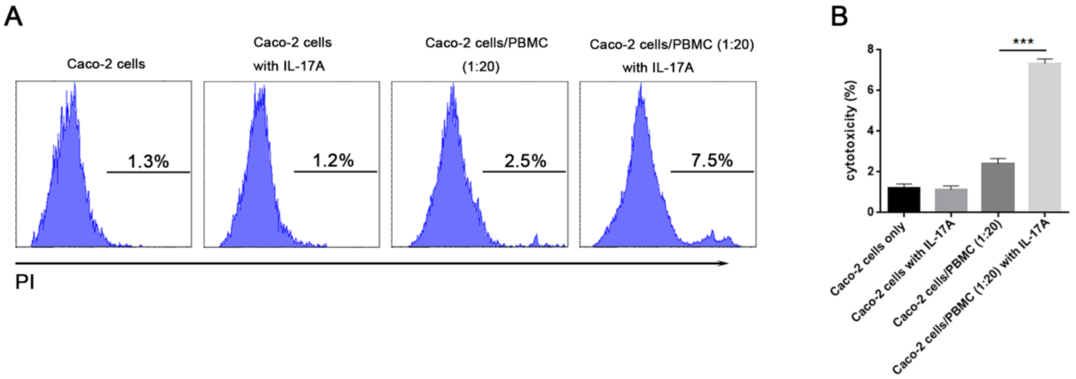

Supplementary Figure 9: Cytotoxic assay revealed that IL-17A increases the cytotoxicity of peripheral blood mononuclear cell (PBMC) against the Caco-2 cells. PBMC from health people were regarded as effector cell, Caco-2 cells were target cell. Caco-2 cells (2×10^4) were labeled with CFSE, then cultured alone or cocultured with PBMC (4×10^5) for 7-hours (with or without recombinant human IL-17A, R&D, 100 ng/ml) in 48-well plate. Staining with Propidium Iodide (PI), the cytotoxicity of PBMC against Caco-2 cells was denoted as the PI-positive rate of Caco-2 cells as analyzed by flow cytometry. **A,** the representative flow cytometric figure of the PI-positive rate of Caco-2 cells. **B,** summary of the cytotoxicity of PBMC against Caco-2 cells, n=6. The PBMC was isolated using density centrifugation on Ficoll from a health man (27 years old), and this was approved by the Ethics Committee of the First Affiliated Hospital, Zhejiang University (Permit number: 2016-267). Caco-2 cells were obtained from Shanghai Cell Bank of Chinese Academy of Sciences and grown in DMEM (Gibco) with 20% FBS (Gibco). Data are expressed as mean ± SEM from three independent experiments.
